# Modelling Malaria Control

**DOI:** 10.1371/journal.pmed.0030111

**Published:** 2006-04-04

**Authors:** Nicholas J White

## Abstract

White discusses a new study that models the impact of deploying intermittent presumptive treatment of malaria upon the spread of drug resistance.

In the past ten years, the rich world has begun to get serious about tackling the diseases that predominantly affect poor people, diseases that impose an enormous humanitarian and economic burden upon those least able to bear it. Infections comprise the majority of this burden, and three have been singled out for particular attention: HIV/AIDS, tuberculosis, and malaria. Of these, malaria is probably the easiest to attack. But how should we attack it?

## Strategies for Malaria Control

Traditionally, we have relied on vector control (killing anopheline mosquitoes) and drug treatment of malaria episodes, and this combined approach has proved remarkably effective in many settings. Malaria is no longer a problem in most of China, Russia, Europe, and North America. But attempts at global eradication foundered in the tropics in the 1960s [ 1], and we have been reluctant to try again.

Waiting for a malaria vaccine has provided one excuse for treading water, but whilst we have waited, malaria morbidity and mortality have worsened [ 2]. Resistance to the widely available and inexpensive chloroquine and sulphadoxine-pyrimethamine (SP) has been the main culprit [ 3]. Doing nothing is not an option. Fortunately, we do now have malaria control interventions that work: effective insecticides, insecticide-impregnated materials (particularly bed nets), and highly effective drugs. And there is increasing political will to direct international donor assistance to malaria control.

## How Should Donor Funds Be Spent?

So how should we use the increasing funds available for purchasing malaria control tools to best effect? In particular, how can we prevent or at least delay losing the new antimalarial drugs to resistance? If we are to roll back malaria, we will certainly need to deploy highly effective antimalarial drugs on a much wider scale than we do now.

There are some areas of broad agreement, many of which apply more generally to infectious disease treatments. Inadequate dosing must be avoided, fixed combinations of drugs (as used in the treatment of tuberculosis and HIV/AIDS) should become the norm, and unnecessary overuse should be minimised. When large numbers of malaria parasites are exposed to antimalarial drugs, then spontaneously arising mutants with point mutations or gene amplifications that confer reduced drug susceptibility may be selected. This selective pressure is inevitable when slowly eliminated drugs are deployed on a large scale in malaria-endemic areas.

How can we prevent or at least delay losing the new antimalarial drugs to resistance?

Resistance has developed to all currently available classes of antimalarial drugs, with the important exception of the artemisinin compounds. Mass treatment and large-scale prophylaxis with antimalarial drugs (mainly chloroquine, pyrimethamine, and piperaquine) has been associated with the emergence of resistance. Chemoprophylaxis is now recommended only for travellers and for women in pregnancy, although the only drugs considered safe enough for continuous use in pregnancy (chloroquine and proguanil) are now largely ineffective against falciparum malaria.

## An Alternative Strategy: Intermittent Presumptive Therapy

An alternative control strategy that has proved effective in reducing the adverse effects of malaria in pregnancy and infancy is to give full treatment doses at intervals [ 4]. This intermittent presumptive treatment (IPT) has been evaluated mainly in areas of high stable transmission, and mainly with SP (and mainly at a time when SP was a lot more effective than it is today). IPT currently involves administering treatment doses in the second and third trimesters of pregnancy, or together with routine childhood immunisations (the Expanded Programme of Immunisation) at two, three, and nine months of age. To date, IPT deployment is limited and is largely confined to SP or amodiaquine as monotherapies, but deployment is likely to increase considerably. However, such extensive deployment of antimalarials to healthy pregnant women and infants would provide a selection pressure to the emergence of drug resistance. It might jeopardise the valuable new drugs now being introduced as treatments. As we cannot measure selection pressure directly, and as a complex mix of interacting factors determine the epidemiology of malaria and antimalarial drug resistance, we must resort to predictive modelling as a guide to possible future outcomes.

Wendy Prudhomme O'Meara and colleagues provide us with such a modelling exercise in a new study in *PLoS Medicine* [ 5]. Their study is a comprehensive one, and it is sophisticated by comparison with earlier modelling exercises in this field. Their model incorporates immunity, transmission intensity, and pharmacokinetic properties of the drugs. Using a simplified binary classification of resistance, they predict that “partially resistant” parasites are more likely to arise in low-transmission areas, but “fully resistant” parasites are more likely to spread under conditions of high transmission. The model predicts that deployment of intermittent preventive treatment in infants could accelerate the spread of resistant parasites, but that this effect would be significant only in areas of low or unstable transmission (where IPT has yet to be deployed). The authors recommend that drugs to which little or no resistance exists should not be used for IPT in high-transmission areas (otherwise, they might be lost prematurely), but that use of IPT in infants is not likely to affect the spread of highly resistant parasites significantly in areas where partial resistance is already established.

## Implications of the New Model

Wendy Prudhomme O'Meara and colleagues' conclusions are generally reassuring. But are they right? More importantly, should we act on the basis of these predictions? For example, should policymakers not deploy new antimalarial drugs, to which resistance has not yet developed (that is, assuming they are safe and effective), for IPT—as recommended by the authors?

The problem with malaria models, even one as advanced and comprehensive as this new model, is that they are still a vast oversimplification of a very complex system. The characterisations of immunity, population antimalarial pharmacokinetics and pharmacodynamics, and the epidemiology of malaria transmission in current models are all insufficient. In addition, geographical, behavioural, and biological heterogeneities are not accounted for adequately. Modellers should be encouraged to describe clearly the limitations of their predictions (based on equations that most general readers studiously avoid attempting to digest). We really do not know whether the results of sensitivity analyses and the conclusions based on these simplifications can be extrapolated to the “real world”. In this particular case, we are not even very sure how IPT “works”.

So we should treat the results of such well-conducted modelling exercises with caution, but we should not ignore them either, as they provide us with valuable direction in research and identify areas of potential concern. In this case, the new model provides an important contribution to a difficult debate that has yet to provide a clear consensus: how and where should IPT be used, and what drugs should be used for it? The modellers need better data to refine their models and to make them more likely to produce realistic results. The debate will go on. Meanwhile, we should not delay in implementing other malaria control measures of proven effectiveness—including deployment of highly efficacious antimalarial combination drug treatment for symptomatic malaria in all malaria-endemic areas.

## Abbreviations

IPT: intermittent presumptive treatment
SP: sulphadoxine-pyrimethamine
